# 3-{[(*E*)-(2-Hydroxynaphthalen-1-yl)methylidene]amino}pyridinium per­chlorate

**DOI:** 10.1107/S1600536813023015

**Published:** 2013-08-21

**Authors:** Maamar Damous, George Dénès, Sofiane Bouacida, Meriem Hamlaoui, Hocine Merazig, Jean-Claude Daran

**Affiliations:** a Unité de Recherche de Chimie de l’Environnement et Moléculaire Structurale, CHEMS, Université Constantine 1, 25000, Algeria; b Laboratory of Solid State Chemistry and Mössbauer Spectroscopy, Laboratories for Inorganic Materials, Department of Chemistry and Biochemistry, Concordia University, Montreal, Quebec, H3G 1M8, Canada; c Département Sciences de la Matière, Faculté des Sciences Exactes et Sciences de la Nature et de la Vie, Université Oum El Bouaghi 04000, Algeria; d Laboratoire de Chimie de Coordination, UPR CNRS 8241, 205 route de Narbonne, 31077 Toulouse cedex, France

## Abstract

In the title Schiff base salt, C_16_H_13_N_2_O^+^·ClO_4_^−^, the pyridine ring and the naphthalene ring system are approximately co-planar [making a dihedral angle of 6.05 (12)°] and an intra­molecular O—H⋯N hydrogen bond occurs between the hydroxyl and imino groups. In the crystal, the cations and anions are linked by N—H⋯O and weak C—H⋯O hydrogen bonds, forming the supra­molecular layers parallel to (100). The crystal studied was an inversion twin refined with minor component = 0.43 (13).

## Related literature

For the pharmaceutical and medicinal activity of Schiff bases, see: Dao *et al.* (2000 ▶); Sriram *et al.* (2006 ▶); Karthikeyan *et al.* (2006 ▶). For the coordination chemistry of Schiff bases, see: Ali *et al.* (2008 ▶); Kargar *et al.* (2009 ▶); Yeap *et al.* (2009 ▶). For the crystal structures of related Schiff base compounds, see: Damous *et al.* (2011 ▶); Fun *et al.* (2009 ▶); Nadeem *et al.* (2009 ▶); Eltayeb *et al.* (2008 ▶). For a description of the Cambridge Structural Database, see: Allen (2002 ▶).
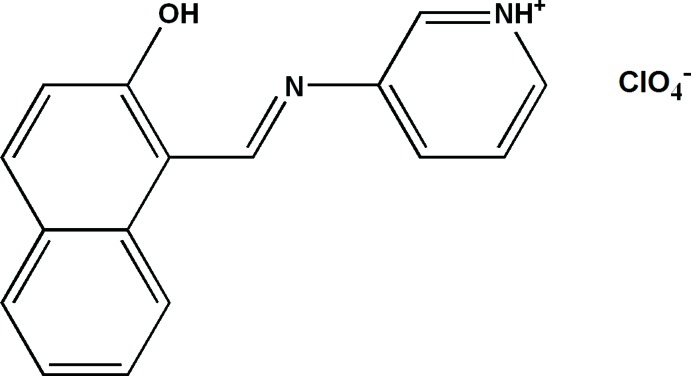


## Experimental

### 

#### Crystal data

C_16_H_13_N_2_O^+^·ClO_4_^−^
*M* *_r_* = 348.73
Orthorhombic, 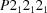

*a* = 6.5043 (9) Å
*b* = 14.6915 (19) Å
*c* = 15.398 (3) Å
*V* = 1471.4 (4) Å^3^
*Z* = 4
Mo *K*α radiation
μ = 0.29 mm^−1^
*T* = 180 K
0.25 × 0.04 × 0.03 mm

#### Data collection

Agilent Xcalibur (Sapphire1) diffractometer
Absorption correction: multi-scan (*CrysAlis PRO*; Agilent, 2011 ▶) *T*_min_ = 0.685, *T*_max_ = 1.000
9582 measured reflections
3369 independent reflections
1695 reflections with *I* > 2σ(*I*)
*R*_int_ = 0.069

#### Refinement

*R*[*F* ^2^ > 2σ(*F* ^2^)] = 0.059
*wR*(*F* ^2^) = 0.127
*S* = 0.92
3369 reflections
218 parameters
H-atom parameters constrained
Δρ_max_ = 0.26 e Å^−3^
Δρ_min_ = −0.28 e Å^−3^
Absolute structure: Flack (1983 ▶), 1362 Friedel pairs
Absolute structure parameter: 0.43 (13)

### 

Data collection: *CrysAlis PRO* (Agilent, 2011 ▶); cell refinement: *CrysAlis PRO*; data reduction: *CrysAlis PRO*; program(s) used to solve structure: *SIR2002* (Burla *et al.*, 2005 ▶); program(s) used to refine structure: *SHELXL97* (Sheldrick, 2008 ▶); molecular graphics: *ORTEP-3 for Windows* (Farrugia, 2012 ▶) and *DIAMOND* (Brandenburg & Berndt, 2001 ▶); software used to prepare material for publication: *WinGX* publication routines (Farrugia, 1012).

## Supplementary Material

Crystal structure: contains datablock(s) global, I. DOI: 10.1107/S1600536813023015/xu5730sup1.cif

Structure factors: contains datablock(s) I. DOI: 10.1107/S1600536813023015/xu5730Isup2.hkl

Additional supplementary materials:  crystallographic information; 3D view; checkCIF report

## Figures and Tables

**Table 1:** Hydrogen-bond geometry (Å, °)

*D*—H⋯*A*	*D*—H	H⋯*A*	*D*⋯*A*	*D*—H⋯*A*
O1—H1⋯N1	0.82	1.82	2.545 (4)	147
N5—H5⋯O13^i^	0.86	2.09	2.844 (5)	146
C2—H2⋯O11	0.93	2.44	3.354 (5)	166
C3—H3⋯O1^ii^	0.93	2.59	3.260 (5)	129
C13—H13⋯O12^iii^	0.93	2.57	3.451 (6)	158

Symmetry codes: (i) 
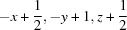
; (ii) 
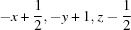
; (iii) 
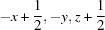
.

**Fig. 1.**
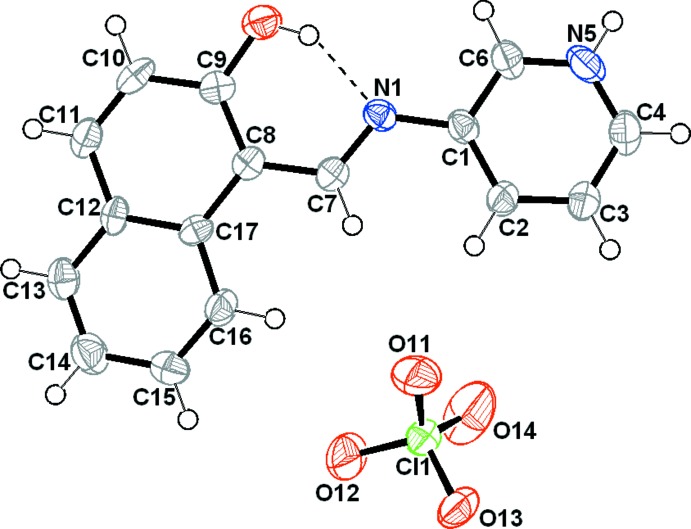
The molecule structure of the title compound with the atomic labelling scheme (Farrugia, 2012). Displacement are drawn at the 50% probability level. H atoms are represented as small spheres of arbitrary radius. Intramolecular hydrogen bond O—H···N is showing as dashed line.

**Fig. 2.**
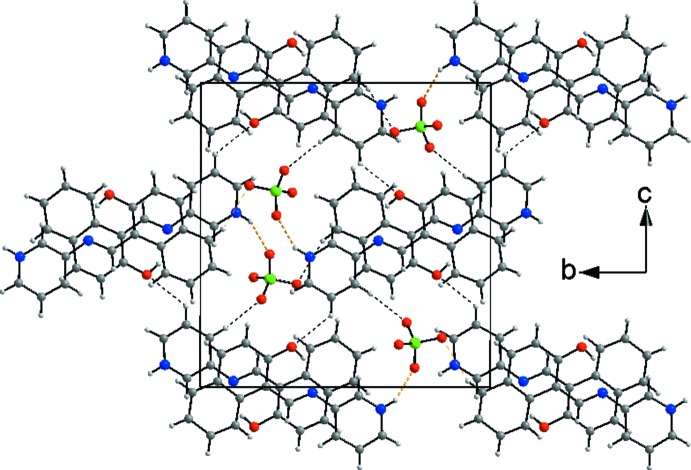
Hydrogen bond connections as dashed line in the same layers parallel to (100) plane between cations and anions (Brandenburg & Berndt, 2001).

**Fig. 3.**
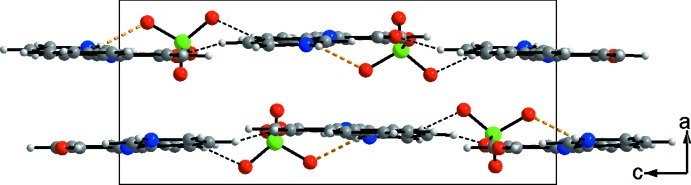
A diagram of the layered crystal packing in (I), viewed down the b axis showing hydrogen bond as dashed line (Brandenburg & Berndt, 2001)

## supplementary crystallographic information

### 1. Comment

Schiff base compounds are a class of important materials used as pharmaceuticals and in various medicinal fields of interest (Dao *et al.*, 2000; Sriram *et al.*, 2006; Karthikeyan *et al.*, 2006). Schiff bases have also been used as versatile ligands in coordination chemistry (Ali *et al.*, 2008; Kargar *et al.*, 2009; Yeap *et al.*, 2009). Recently, the crystal structures of a large number of new Schiff base compounds have been reported (Fun *et al.*, 2009; Nadeem *et al.*, 2009; Eltayeb *et al.*, 2008; Damous *et al.*, 2011).

Herein we report the synthesis and crystal structure of 3-{[*E*)-(2-Hydroxynaphthalen-1-yl)methylidene]amino}pyridinium perchlorate, (I). The molecular structure of (I), and the atomic numbering used, is illustrated in Fig. 1. The asymmetric unit of (I) consists of one protoned *N*-(3-pyridil)-2-oxo-1-naphthylidenemethylamine cation and one perchlorate anion. All bond distances and angles are within the ranges of accepted values(CSD, Allen, 2002). The cation is co-planar with r.m.s. deviation all non-H atoms, for cation, are essentially co-planar with a maximum deviation of -0.1158 (39) Å for N5 [r.m.s. deviation: 0.0590 Å]. The molecule is twisted with the dihedral angle between the benzene and the naphthyl ring mean planes being 6.05 (12) °.

In the crystal structure, cationic and anionic layers alternate along the *c* axis and are linked by intermolecular N—H···O and weak C—H···O hydrogen bonds (Table 1, Fig.2) resulting in a two-dimensional network parallel to (100)plane (Fig.3). Also, we observe an intramolecular O—H···N hydrogen bond, involving the naphthalene hydroxyl substituent and the pyridine N-atom (Fig.1, Table 1).

### 2. Experimental

The title compound, (I), was prepared by refluxing a mixture of a solution containing (0.1 mmol) of 2-hydroxy-1-naphthaldehyde and (0.1 mmol) of 3-aminopyridine in presence of perchloric acid in 20 ml methanol. The reaction mixture was stirred for 1 h under reflux. Microcrystals of (I) were obtained by allowing the clear solution to stand overnight. The powder product was dissolved and recrystallized from DMSO solution. Some red crystals were carefully isolated under polarizing microscope for analysis by X-ray diffraction.

### 3. Refinement

All non-H atoms were refined with anisotropic atomic displacement parameters. The remaining H atoms were localized on Fourier maps but introduced in calculated positions and treated as riding on their parent atoms (C, O and N) with C—H = 0.93 Å, N—H = 0.86 Å and O—H = 0.82 Å and *U*_iso_(H)=1.2*U*_eq_(C or N); *U*_iso_(H)=1.5*U*_eq_(O).

The crystal used is an inversion twin with refined components 0.57 and 0.43.

### Crystal data

**Table:**

C_16_H_13_N_2_O^+^·ClO_4_^−^	*D*_x_ = 1.574 Mg m^−^^3^
*M**_r_* = 348.73	Mo *K*α radiation, λ = 0.71073 Å
Orthorhombic, *P*2_1_2_1_2_1_	Cell parameters from 1867 reflections
*a* = 6.5043 (9) Å	θ = 3.0–28.4°
*b* = 14.6915 (19) Å	µ = 0.29 mm^−^^1^
*c* = 15.398 (3) Å	*T* = 180 K
*V* = 1471.4 (4) Å^3^	Needle, red
*Z* = 4	0.25 × 0.04 × 0.03 mm
*F*(000) = 720	

### Data collection

**Table:**

Agilent Xcalibur (Sapphire1) diffractometer	3369 independent reflections
Radiation source: fine-focus sealed tube	1695 reflections with *I* > 2σ(*I*)
Graphite monochromator	*R*_int_ = 0.069
Detector resolution: 8.2632 pixels mm^-1^	θ_max_ = 28.5°, θ_min_ = 3.0°
ω scans	*h* = −8→8
Absorption correction: multi-scan (*CrysAlis PRO*; Agilent, 2011)	*k* = −19→19
*T*_min_ = 0.685, *T*_max_ = 1.000	*l* = −19→19
9582 measured reflections	

### Refinement

**Table:**

Refinement on *F*^2^	Secondary atom site location: difference Fourier map
Least-squares matrix: full	Hydrogen site location: inferred from neighbouring sites
*R*[*F*^2^ > 2σ(*F*^2^)] = 0.059	H-atom parameters constrained
*wR*(*F*^2^) = 0.127	*w* = 1/[σ^2^(*F*_o_^2^) + (0.0494*P*)^2^] where *P* = (*F*_o_^2^ + 2*F*_c_^2^)/3
*S* = 0.92	(Δ/σ)_max_ < 0.001
3369 reflections	Δρ_max_ = 0.26 e Å^−^^3^
218 parameters	Δρ_min_ = −0.28 e Å^−^^3^
0 restraints	Absolute structure: Flack (1983), 1362 Friedel pairs
Primary atom site location: structure-invariant direct methods	Absolute structure parameter: 0.43 (13)

### Special details

**Table:**

Geometry. All e.s.d.'s (except the e.s.d. in the dihedral angle between two l.s. planes) are estimated using the full covariance matrix. The cell e.s.d.'s are taken into account individually in the estimation of e.s.d.'s in distances, angles and torsion angles; correlations between e.s.d.'s in cell parameters are only used when they are defined by crystal symmetry. An approximate (isotropic) treatment of cell e.s.d.'s is used for estimating e.s.d.'s involving l.s. planes.
Refinement. Refinement of *F*^2^ against ALL reflections. The weighted *R*-factor *wR* and goodness of fit *S* are based on *F*^2^, conventional *R*-factors *R* are based on *F*, with *F* set to zero for negative *F*^2^. The threshold expression of *F*^2^ > σ(*F*^2^) is used only for calculating *R*-factors(gt) *etc*. and is not relevant to the choice of reflections for refinement. *R*-factors based on *F*^2^ are statistically about twice as large as those based on *F*, and *R*- factors based on ALL data will be even larger.

### Fractional atomic coordinates and isotropic or equivalent isotropic displacement parameters (Å^2^)

**Table:**

	*x*	*y*	*z*	*U*_iso_*/*U*_eq_	
Cl1	0.26988 (16)	0.25979 (7)	0.14441 (7)	0.0420 (3)	
O1	0.3025 (4)	0.31465 (18)	0.63157 (19)	0.0404 (7)	
H1	0.3022	0.3556	0.5954	0.061*	
O13	0.3797 (5)	0.2618 (2)	0.0636 (2)	0.0567 (9)	
O12	0.2121 (6)	0.1681 (2)	0.1622 (2)	0.0702 (10)	
N1	0.2985 (5)	0.3865 (2)	0.4812 (2)	0.0283 (9)	
O11	0.4044 (5)	0.2891 (2)	0.2118 (2)	0.0647 (10)	
C17	0.2947 (6)	0.1364 (2)	0.4624 (3)	0.0271 (10)	
C8	0.2991 (5)	0.2266 (2)	0.5012 (2)	0.0267 (9)	
C9	0.3004 (5)	0.2340 (3)	0.5911 (3)	0.0297 (10)	
C16	0.2929 (5)	0.1213 (3)	0.3737 (3)	0.0357 (10)	
H16	0.2964	0.1707	0.3359	0.043*	
C10	0.2994 (6)	0.1562 (3)	0.6438 (3)	0.0366 (10)	
H10	0.3015	0.1627	0.7039	0.044*	
C12	0.2913 (6)	0.0598 (2)	0.5180 (3)	0.0293 (9)	
N5	0.2590 (6)	0.6228 (2)	0.4274 (3)	0.0455 (10)	
H5	0.2496	0.6728	0.4562	0.055*	
C1	0.2895 (6)	0.4649 (2)	0.4284 (3)	0.0302 (10)	
C7	0.2958 (6)	0.3064 (2)	0.4483 (3)	0.0284 (9)	
H7	0.2915	0.3	0.3882	0.034*	
C11	0.2954 (6)	0.0720 (3)	0.6088 (3)	0.0373 (11)	
H11	0.2954	0.0214	0.6451	0.045*	
O14	0.0992 (5)	0.3161 (3)	0.1393 (3)	0.1075 (16)	
C2	0.2884 (6)	0.4676 (3)	0.3384 (3)	0.0360 (11)	
H2	0.2999	0.4138	0.307	0.043*	
C13	0.2841 (7)	−0.0278 (3)	0.4825 (3)	0.0390 (11)	
H13	0.2807	−0.0781	0.5192	0.047*	
C3	0.2706 (7)	0.5487 (3)	0.2955 (3)	0.0405 (11)	
H3	0.2684	0.5499	0.2351	0.049*	
C4	0.2562 (7)	0.6270 (3)	0.3411 (3)	0.0443 (11)	
H4	0.2445	0.6827	0.3127	0.053*	
C15	0.2861 (7)	0.0350 (3)	0.3406 (3)	0.0455 (12)	
H15	0.2843	0.0265	0.2807	0.055*	
C6	0.2756 (7)	0.5459 (2)	0.4712 (3)	0.0374 (11)	
H6	0.2778	0.5469	0.5316	0.045*	
C14	0.2819 (7)	−0.0405 (3)	0.3954 (3)	0.0471 (12)	
H14	0.2777	−0.099	0.3724	0.056*	

### Atomic displacement parameters (Å^2^)

**Table:**

	*U*^11^	*U*^22^	*U*^33^	*U*^12^	*U*^13^	*U*^23^
Cl1	0.0533 (6)	0.0401 (6)	0.0327 (6)	0.0063 (6)	0.0004 (6)	0.0032 (5)
O1	0.0468 (17)	0.0434 (16)	0.0311 (19)	−0.0026 (14)	0.0021 (18)	−0.0021 (14)
O13	0.078 (2)	0.059 (2)	0.033 (2)	0.0139 (19)	0.0153 (16)	0.0113 (18)
O12	0.106 (3)	0.0500 (19)	0.055 (3)	−0.036 (2)	0.000 (3)	0.0080 (16)
N1	0.027 (2)	0.0273 (17)	0.030 (2)	−0.0024 (16)	0.0026 (17)	−0.0030 (15)
O11	0.089 (2)	0.057 (2)	0.048 (3)	−0.0196 (19)	−0.006 (2)	−0.0124 (19)
C17	0.021 (2)	0.036 (2)	0.024 (3)	−0.0001 (19)	−0.003 (2)	−0.0003 (18)
C8	0.0181 (18)	0.034 (2)	0.028 (2)	0.0023 (18)	0.0019 (18)	0.0030 (18)
C9	0.023 (2)	0.036 (2)	0.030 (3)	−0.003 (2)	0.0009 (18)	−0.0020 (19)
C16	0.040 (2)	0.036 (2)	0.031 (3)	−0.005 (2)	0.004 (2)	0.0080 (18)
C10	0.033 (2)	0.055 (3)	0.022 (2)	−0.001 (2)	0.001 (2)	0.009 (2)
C12	0.023 (2)	0.030 (2)	0.034 (3)	−0.0001 (19)	0.001 (2)	0.0113 (18)
N5	0.056 (3)	0.0278 (19)	0.052 (3)	−0.005 (2)	0.006 (2)	−0.0102 (17)
C1	0.031 (2)	0.025 (2)	0.035 (3)	−0.004 (2)	0.003 (2)	−0.0012 (18)
C7	0.023 (2)	0.033 (2)	0.029 (2)	−0.0020 (19)	0.004 (2)	0.0023 (19)
C11	0.038 (2)	0.039 (2)	0.034 (3)	0.006 (2)	0.003 (2)	0.012 (2)
O14	0.104 (3)	0.128 (3)	0.091 (4)	0.078 (3)	0.039 (3)	0.043 (3)
C2	0.046 (3)	0.031 (2)	0.031 (3)	0.004 (2)	0.008 (2)	0.0033 (19)
C13	0.039 (3)	0.032 (2)	0.047 (3)	−0.003 (2)	0.004 (3)	0.009 (2)
C3	0.050 (3)	0.034 (2)	0.037 (3)	0.002 (3)	0.003 (3)	0.007 (2)
C4	0.049 (3)	0.032 (2)	0.051 (4)	0.001 (2)	0.006 (3)	0.010 (2)
C15	0.060 (3)	0.038 (3)	0.038 (3)	−0.002 (3)	0.002 (3)	−0.008 (2)
C6	0.041 (3)	0.027 (2)	0.044 (3)	−0.002 (2)	0.003 (3)	0.0008 (19)
C14	0.054 (3)	0.032 (2)	0.055 (3)	−0.005 (3)	0.005 (3)	0.000 (2)

### Geometric parameters (Å, º)

**Table:**

Cl1—O14	1.387 (3)	C12—C11	1.411 (5)
Cl1—O11	1.424 (3)	N5—C6	1.321 (5)
Cl1—O12	1.425 (3)	N5—C4	1.331 (5)
Cl1—O13	1.436 (3)	N5—H5	0.86
O1—C9	1.339 (4)	C1—C6	1.363 (5)
O1—H1	0.82	C1—C2	1.386 (5)
N1—C7	1.282 (4)	C7—H7	0.93
N1—C1	1.411 (5)	C11—H11	0.93
C17—C16	1.384 (6)	C2—C3	1.367 (5)
C17—C12	1.414 (5)	C2—H2	0.93
C17—C8	1.455 (5)	C13—C14	1.353 (6)
C8—C9	1.388 (5)	C13—H13	0.93
C8—C7	1.428 (5)	C3—C4	1.351 (5)
C9—C10	1.402 (5)	C3—H3	0.93
C16—C15	1.367 (5)	C4—H4	0.93
C16—H16	0.93	C15—C14	1.394 (5)
C10—C11	1.349 (5)	C15—H15	0.93
C10—H10	0.93	C6—H6	0.93
C12—C13	1.399 (5)	C14—H14	0.93
			
O14—Cl1—O11	110.6 (3)	C6—C1—N1	115.9 (4)
O14—Cl1—O12	111.3 (3)	C2—C1—N1	126.8 (3)
O11—Cl1—O12	107.9 (2)	N1—C7—C8	121.9 (4)
O14—Cl1—O13	109.7 (2)	N1—C7—H7	119.1
O11—Cl1—O13	108.6 (2)	C8—C7—H7	119.1
O12—Cl1—O13	108.5 (2)	C10—C11—C12	120.9 (4)
C9—O1—H1	109.5	C10—C11—H11	119.6
C7—N1—C1	121.4 (4)	C12—C11—H11	119.6
C16—C17—C12	118.0 (4)	C3—C2—C1	120.6 (4)
C16—C17—C8	123.5 (4)	C3—C2—H2	119.7
C12—C17—C8	118.5 (4)	C1—C2—H2	119.7
C9—C8—C7	120.3 (4)	C14—C13—C12	120.9 (4)
C9—C8—C17	118.8 (3)	C14—C13—H13	119.5
C7—C8—C17	120.9 (4)	C12—C13—H13	119.5
O1—C9—C8	122.2 (3)	C4—C3—C2	119.8 (4)
O1—C9—C10	116.9 (4)	C4—C3—H3	120.1
C8—C9—C10	120.9 (4)	C2—C3—H3	120.1
C15—C16—C17	121.1 (4)	N5—C4—C3	118.6 (4)
C15—C16—H16	119.4	N5—C4—H4	120.7
C17—C16—H16	119.4	C3—C4—H4	120.7
C11—C10—C9	121.1 (4)	C16—C15—C14	120.8 (4)
C11—C10—H10	119.4	C16—C15—H15	119.6
C9—C10—H10	119.4	C14—C15—H15	119.6
C13—C12—C11	120.4 (4)	N5—C6—C1	120.4 (4)
C13—C12—C17	119.7 (4)	N5—C6—H6	119.8
C11—C12—C17	119.9 (4)	C1—C6—H6	119.8
C6—N5—C4	123.4 (4)	C13—C14—C15	119.3 (4)
C6—N5—H5	118.3	C13—C14—H14	120.3
C4—N5—H5	118.3	C15—C14—H14	120.3
C6—C1—C2	117.3 (4)		
			
C16—C17—C8—C9	179.9 (4)	C9—C8—C7—N1	1.6 (6)
C12—C17—C8—C9	0.2 (5)	C17—C8—C7—N1	179.7 (3)
C16—C17—C8—C7	1.8 (6)	C9—C10—C11—C12	−0.2 (6)
C12—C17—C8—C7	−177.9 (4)	C13—C12—C11—C10	−179.0 (4)
C7—C8—C9—O1	−1.3 (5)	C17—C12—C11—C10	1.0 (6)
C17—C8—C9—O1	−179.4 (3)	C6—C1—C2—C3	−1.0 (7)
C7—C8—C9—C10	178.7 (4)	N1—C1—C2—C3	177.3 (4)
C17—C8—C9—C10	0.6 (5)	C11—C12—C13—C14	−179.4 (4)
C12—C17—C16—C15	0.6 (6)	C17—C12—C13—C14	0.5 (6)
C8—C17—C16—C15	−179.2 (4)	C1—C2—C3—C4	0.7 (7)
O1—C9—C10—C11	179.4 (3)	C6—N5—C4—C3	0.1 (8)
C8—C9—C10—C11	−0.6 (6)	C2—C3—C4—N5	−0.2 (7)
C16—C17—C12—C13	−0.7 (6)	C17—C16—C15—C14	−0.3 (6)
C8—C17—C12—C13	179.1 (3)	C4—N5—C6—C1	−0.5 (7)
C16—C17—C12—C11	179.3 (4)	C2—C1—C6—N5	0.9 (7)
C8—C17—C12—C11	−1.0 (6)	N1—C1—C6—N5	−177.6 (4)
C7—N1—C1—C6	174.5 (4)	C12—C13—C14—C15	−0.3 (7)
C7—N1—C1—C2	−3.8 (7)	C16—C15—C14—C13	0.2 (7)
C1—N1—C7—C8	−178.2 (4)		

### Hydrogen-bond geometry (Å, º)

**Table:**

*D*—H···*A*	*D*—H	H···*A*	*D*···*A*	*D*—H···*A*
O1—H1···N1	0.82	1.82	2.545 (4)	147
N5—H5···O13^i^	0.86	2.09	2.844 (5)	146
C2—H2···O11	0.93	2.44	3.354 (5)	166
C3—H3···O1^ii^	0.93	2.59	3.260 (5)	129
C13—H13···O12^iii^	0.93	2.57	3.451 (6)	158

Symmetry codes: (i) −*x*+1/2, −*y*+1, *z*+1/2; (ii) −*x*+1/2, −*y*+1, *z*−1/2; (iii) −*x*+1/2, −*y*, *z*+1/2.
